# A Novel Indicator Based on Polyacrylamide Hydrogel and Bromocresol Green for Monitoring the Total Volatile Basic Nitrogen of Fish

**DOI:** 10.3390/foods12213964

**Published:** 2023-10-30

**Authors:** Zhepeng Zhang, Haiqing Tang, Keyan Cai, Ruiping Liang, Li Tong, Changrong Ou

**Affiliations:** 1 College of Food and Pharmaceutical Sciences, Ningbo University, Ningbo 315832, China; zzp330683@163.com (Z.Z.); caikeyan1121@163.com (K.C.); liangruiping2022@163.com (R.L.); 18888647607@163.com (L.T.); 2 Faculty of Food Science, Zhejiang Pharmaceutical University, Ningbo 315100, China; tanghq@mail.zjpc.net.cn

**Keywords:** TVB-N, polyacrylamide hydrogel, bromocresol green, colorimetric indicator, intelligent packaging

## Abstract

An intelligent indicator was developed by immobilizing bromocresol green (BCG) within the polyacrylamide (PAAm) hydrogel matrix to monitor the total volatile basic nitrogen (TVB-N) content of fish. The FTIR analysis indicated that BCG was effectively incorporated into the PAAm through the formation of intermolecular hydrogen bonds. A thermogravimetric analysis (TGA) showed that the PAAm/BCG indicator had a mere 0.0074% acrylamide monomer residue, meanwhile, the addition of BCG improved the thermal stability of the indicator. In vapor tests with various concentrations of trimethylamine, the indicator performed similarly at both 4 °C and 25 °C. The total color difference values (Δ*E*) exhibited a significant linear response to TVB-N levels ranging from 4.29 to 30.80 mg/100 g at 4 °C (R^2^ = 0.98). Therefore, the PAAm/BCG indicator demonstrated stable and sensitive color changes based on pH variations and could be employed in smart packaging for real-time assessment of fish freshness.

## 1. Introduction

Affected by microorganisms and endogenous enzymes, fish are highly susceptible to spoilage during storage, processing, and transportation. In order to ensure food safety and avoid huge economic losses, real-time monitoring of fish freshness is crucial for consumers and food production enterprises. Recently, the increasing demand to provide high-quality fresh food has led to the emergence of intelligent packaging for monitoring food quality or freshness (such as some small molecule metabolites) using responsive color-changing indicators [1,2]. Freshness indicators work by monitoring or reacting with metabolites such as organic acids, CO_2_, total volatile basic nitrogen (TVB-N) or sulfur derivatives produced in fresh food [3,4,5,6].

The types of freshness indicators are roughly divided into pH-sensitive, microbial-sensitive, hydrogen sulfide-sensitive, and ethylene-sensitive, of which, pH-sensitive indicators are the most commonly studied. Generally speaking, the pH-sensitive indicator consists of a solid substrate and one or more pH-sensitive pigments [7]. Previous studies have shown that certain pH-sensitive pigments, such as anthocyanin, curcumin or alizarin, perform well in colorimetric fish freshness indicators [8,9,10,11]. However, natural pigments are often greatly affected by the environment, including poor antioxidant properties and low melting points, which could cause the pigments to become inactive, thereby leading to the malfunction of intelligent systems. Additionally, the extraction process of this particular type of dye was complex, leading to a minimal overall economic advantage. Hence, as long as the dosage of the synthetic dye was well controlled and overflowing from the matrix was prevented, it had a promising development prospect in large-scale production indicators. Among them, one example of a synthetic pH dye was bromocresol green (BCG), which was highly sensitive to alkaline substances. BCG underwent an immediate color change from yellow to blue when the pH of the external environment slightly increased. In addition, BCG has several advantages over natural colorants, including excellent stability, versatility, and low cost.

Normally, pigments are developed by using hydrophilic membranes as the solid matrix, since colorimetric indicators typically work through pH changes caused by the dissolution of the target substance in water [12,13]. However, poor hygroscopicity of the substrate or limited humidity within the package may result in a reduced sensitivity of the indicator. Since the humidity can affect the sensitivity and effectiveness of the color change, therefore highly hydrophilic or water-containing materials such as hydrogels are typically recommended in indicator preparation to improve the color response of pH-sensitive pigments to target substances generated during fish spoilage.

Hydrogels have interesting biological properties such as softness, wettability, responsiveness, as well as biocompatibility, which can be applied to sensors, actuators, coatings, optics, electronics, and water harvesters [14]. Under the above circumstances, the hydrogel can serve as an inert matrix to accommodate responsive elements that are physically encapsulated in or chemically anchored to the hydrogel matrix, which is permeable to chemical input properties, thereby allowing coactions between external substances and targeting elements [15]. Due to the polyacrylamide (PAAm) hydrogel structural unit containing amide groups, which can easily form hydrogen bonds, it has good colloid stability and hydrophilicity [16]. Currently, a variety of pH-sensitive indicators for the real-time monitoring of fish freshness have been reported, and the results have been encouraging [17,18,19]. However, most of these indicators rely on a complex preparation process, including extraction and purification by organic reagents, adjusting the pH of hydrosol, time-consuming magnetic stirring and hot-air drying [5,9,20]. In contrast, the PAAm/BCG indicator is based on a two-step molding and demolding method using polytetrafluoroethylene mold, which can reduce the preparation cost, simplify the preparation process, and eliminate interference from external factors. In addition, since the aqueous solution of acrylamide is weakly acidic, there was no need to adjust the pH of the solution. Therefore, the PAAm hydrogel has great potential as a solid matrix for pH-sensitive dyes to assess fish freshness. However, few related studies have been reported so far.

Herein, the aim was to develop an indicator using the PAAm hydrogel, as a smart packaging feature for monitoring the freshness of aquatic products. The PAAm hydrogel serves as a carrier for the pH-sensitive pigment BCG, and an absorbent reservoir for volatile amines, to improve the color-changing sensitivity of the indicator. The properties of the PAAm hydrogel/BCG indicator include moisture sensitivity, temperature sensitivity, structural properties, BCG release behavior, as well as evaluating the color response of different pH values and trimethylamine (TMA) vapor. Furthermore, the indicator was also tentatively applied to evaluate sea bass freshness.

## 2. Materials and Methods

### 2.1. Material

BCG was obtained from Sigma-Aldrich (St. Louis, MO, USA). Trimethylamine (TMA), acrylamide and ammonium persulphate were purchased from Aladdin-Reagent Co. (Shanghai, China). Citric Acid, sodium citrate, potassium dihydrogen phosphate, sodium phosphate dibasic anhydrous, ethanol (99.9%), hydrochloric acid, potassium chloride (KCl), magnesium oxide (MgO) and boric acid (H_3_BO_3_) were gained from Chemical Reagent Co., Ltd. (Shanghai, China). All chemicals were analytical grade.

### 2.2. UV-Vis Spectra Characterization of BCG Solution

Configuring several buffers with different pH values, including pH 3.0, 4.0 and 5.0 were created using a citric acid sodium citrate buffer solution and pH 6.0, 7.0 and 8.0 were created using a phosphate buffer solution. The light absorption spectra of BCG solutions (2.0 g/L ethanol) at pH 3.0–8.0 were gained in the scope of 400–800 nm using a UV-vis spectrophotometer (TU-1810, Ningbo OP Instrument Co., Ltd., Ningbo, China).

### 2.3. Preparation of the PAAm Hydrogel/BCG Indicator

The BCG ethanol solution (2.0 g/L) was prepared by dissolving 0.1 g of BCG powder in 50.0 mL of 99.9% ethanol. To prepare the PAAm hydrogel/BCG indicator, 1.0 g acrylamide, 0.02 g ammonium persulphate and 0.2 mL BCG ethanol solution were added to 2.0 mL distilled water. After the solid was completely dissolved by an ultrasonic instrument, the mixed solution was injected into the polytetrafluoroethylene mold and was heated for 25 min at 60 °C. After the indicator was gently peeled out of the mold, it was stored in an airtight container overnight.

### 2.4. Characterization and Properties

#### 2.4.1. Fourier-Transform Infrared (FT-IR) Spectroscopy

To study coactions between the functional groups of PAAm hydrogel and BCG in the structure of the fabricated indicator, the FT-IR measurement of the PAAm hydrogel and the PAAm/BCG indicator were performed on a Thermo Nicolet spectrometer (Nicolet 380, Thermos Scientific Inc., Waltham, MA, USA) in the scope of 4000–500 cm^−1^.

#### 2.4.2. Thermogravimetric Analysis (TGA)

The thermal stability of the indicator was determined using a thermogravimetric analyzer (TGA 209F1, NETZSCH Instrument, Selb, Germany). Detection of TG was based on the method of Roy et al. and had been appropriately modified [21]. We placed 5.0 mg of hydrogel sample in a standard crucible. The samples were heated from 25 to 600 °C at 10 °C/min under nitrogen flow (20 cm^3^/min) with an empty cup as a reference.

#### 2.4.3. Moisture Content (MC), Water Solubility (WS), and Water Absorption (WA)

Detection of MC, WS, and WA was based on the method of Ezati et al. [22] with modification. The MC of the indicator was determined gravimetrically. For each PAAm hydrogel sample and the PAAm/BCG indicator sample, three specimens (Φ 1.2 cm, δ 0.2 cm) were dried at 105 °C for 2 h with an Instrument for Moisture Determination. The weight of the samples before drying (M_1_) and after drying (M_2_) was measured, respectively. For the determination of WS, three dried samples (Φ 1.2 cm, δ 0.2 cm) were weighed (M_2_) and then immersed in a beaker containing 50.0 mL distilled water for 2 h with mild stirring. The samples were removed from the beaker and dried at 105 °C for 2 h and weighed (M_3_). The indicator samples (Φ 1.2 cm, δ 0.2 cm) were immersed in distilled water at 25 °C for 1 h to measure the WA of the indicator. Collect the samples and remove surface water with blotting paper. WA was calculated by measuring the percentage increase in indicator weight before (M_1_) and after immersion (M_4_). The MC, WS, and WA of the samples were calculated as follows:

(1) $$MC\left(\%\right)=\frac{M_{1}-M_{2}}{M_{1}}\times 100$$

(2) $$WS\;\left(\%\right)=\frac{M_{2}-M_{3}}{M_{2}}\times 100$$

(3) $$WA\;\left(\%\right)=\frac{M_{4}-M_{1}}{M_{1}}\times 100$$

#### 2.4.4. Release of BCG

The release of BCG from the indicator was determined using food simulators (95%, 50%, and 10% ethanol and water) [23]. Among them, the three food simulants containing alcohol represent high-fat food, oil-in-water emulsion food, and water-containing food respectively. For this, an indicator (Φ 1.2 cm, δ 0.2 cm) was added to a conical centrifuge tube containing 20.0 mL of the food simulant and kept at 25 ℃ with gentle shaking. The releasing rate for the aforementioned indicator was determined by the absorbance at 615 nm using a UV-vis spectrophotometer at time intervals of 0, 6, 12, 36, 48, and 72 h.

### 2.5. Color Response Efficiency

The color response efficiency detection of the PAAm hydrogel/BCG indicator was modified based on the method of Ezati et al. [24]. The indicator (Φ 1.2 cm, δ 0.2 cm) was soaked into the citric acid sodium citrate and phosphate buffer solution for 10 min at 25 °C. The Chroma meter (NR110, Shenzhen 3NH Technology Co., Ltd., Shenzhen, China) was used to determine tristimulus color values (standard white plate: L = 96.52, a = −0.24, and b = 0.46). The total color difference (Δ*E*) was calculated as follows:

(4) $$\Delta E=\sqrt{{\left(\Delta L\right)}^{2}+{\left(\Delta a\right)}^{2}+{\left(\Delta b\right)}^{2}}$$

where Δ*L*, Δ*a*, and Δ*b* are the difference between each value of the standard color plate and indicator, respectively.

The indicator was hung over a sealed centrifuge tube with different concentrations of TMA solution for 10 min at 25 °C and 4 °C, respectively, and we recorded the tristimulus color values to assess the sensitivity of the indicator for alkaline gas and the influence of temperature on the indicator.

### 2.6. Packaging Test

The PAAm hydrogel/BCG indicator was used in fish packaging to monitor freshness change during storage. Live Japanese sea bass (*Lateolabrax japonicus*) were purchased from a local market (Ningbo, China), slaughtered immediately after transport to the laboratory, skinned, and deboned. We put 30.0 g of fish meat into each polypropylene container (16.8 cm × 11.7 cm × 3.0 cm) for freshness monitoring. Since the indicator has a certain adhesiveness, it can be directly attached to the headspace of the container. Experiments were performed under refrigeration (4 °C), and from 0 d, TVB-N levels, pH, and tristimulus color values of the indicator were recorded every other day.

#### 2.6.1. TVB-N Measurement

An aliquot of 2 g homogenized fish muscle was weighed and placed into a centrifuge tube with 20 mL of deionized water, and then treated with a homogenizer for 1 min to homogenize the fish sample in the centrifuge tube. Then, 5.0 mL of MgO suspension (10 g/L) was added to the distillation tube and mixed well with the homogenized sample in the centrifuge tube. Finally, the distillation tube was connected to a K9840 fully automated Kjeldahl analyzer (Shandong, China), and automatic analysis was performed. The analysis was repeated in triplicate.

#### 2.6.2. Determination of pH Values

For pH values determination, an aliquot of 2 g fish muscle and 20.0 mL of 0.1 mol/L KCl were placed in a centrifuge tube and we homogenized the sample with a homogenizer at 25 ℃. The above mixture was analyzed using a pH meter (PHS-2F, Leici, China), which was repeated in triplicate.

### 2.7. Statistical Analysis

All data were analyzed using analysis of variance (ANOVA) in SPSS v 25.0.0 (SPSS Inc., Chicago, IL, USA) and expressed as mean ± standard deviation. All results were considered statistically significant at *p*-values < 0.05.Interventional studies involving animals or humans, and other studies that require ethical approval, must list the authority that provided approval and the corresponding ethical approval code.

## 3. Results and Discussion

### 3.1. UV-Vis Spectra Analysis

**Figure 1:**
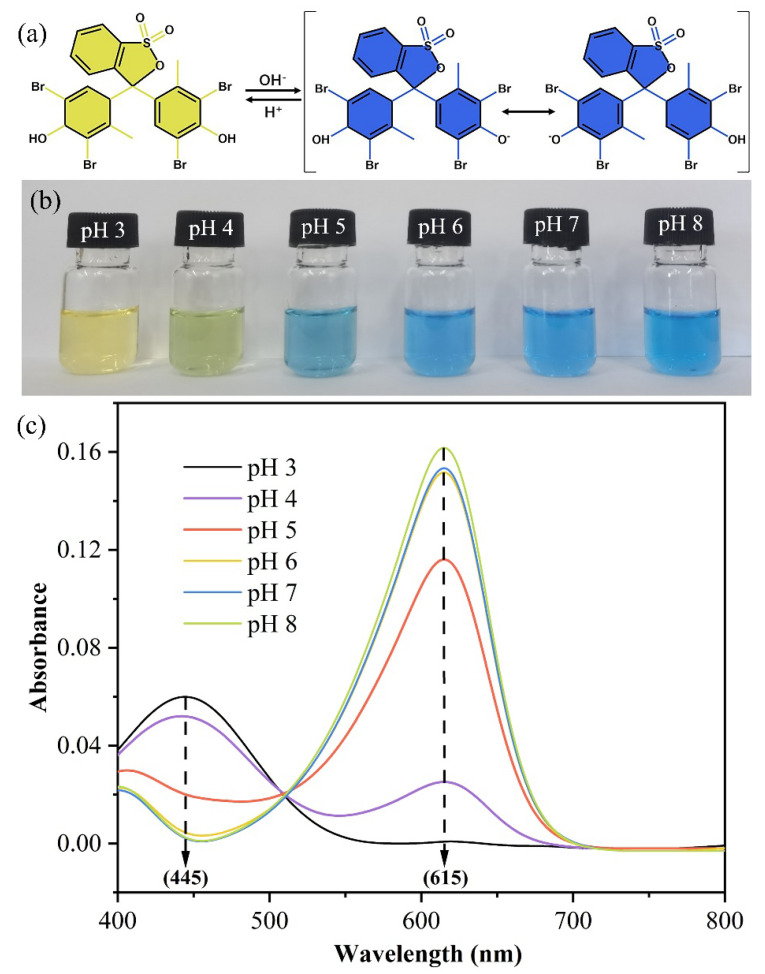
(**a**) Principle schematic diagram of color change of BCG dye at acid base conditions. (**b**) The color of BCG solution at various pH from 3.0 to 8.0. (**c**) UV-vis spectra of BCG solution at various pH from 3.0 to 8.0.

As shown in Figure 1a, BCG was a sulfonphthalein pigment, with a pH transition from 3.8 to 5.4. In its acidic form, this dye appears brightly yellow, and at alkaline pH, the color becomes blue. Moreover, in the aqueous solution, BCG ionizes to the monoanionic form (yellow), which was then deprotonated at a higher pH to a form resonance-stabilized dianionic form (blue) [25].

The discoloration properties and corresponding UV-vis absorption spectra of BCG solution at different pH values were tested. Figure 1b shows the BCG solution was bright yellow at pH 3.0 and became dark with the pH increased, turning blue at pH 5.0, and then the blue deepeneds gradually. Accordingly, corresponding to the maximum absorbance capacity also changed in the following analysis. The maximum absorption peak of BCG shifted from 445 nm to 615 nm as pH was raised (Figure 1c), and the maximum peak size increased with the increase in solution alkalinity [26]. The intense color change indicates that BCG was very sensitive to basic compounds. Therefore, it was suitable for manufacturing colorimetric sensing indicators for fish freshness monitoring.

### 3.2. Properties of the Indicator

#### 3.2.1. FTIR Characterization

**Figure 2:**
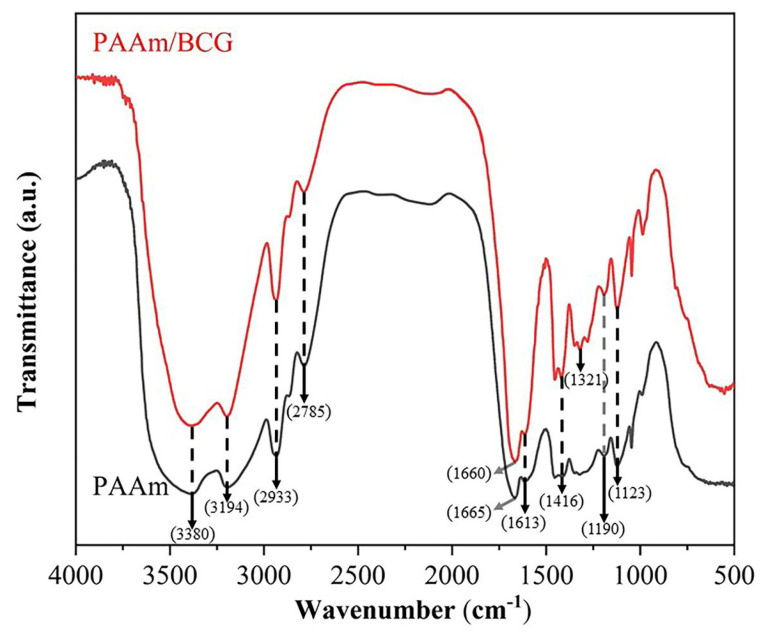
FTIR spectra of the PAAm hydrogel and PAAm/BCG indicator.

The FTIR spectra of PAAm hydrogel and indicator samples are presented in Figure 2, including the peaks at 3380 cm^−1^, 3194 cm^−1^, and 1613 cm^−1^ corresponding to the antisymmetric stretching vibration, symmetric stretching vibration, and stretching vibration of N-H, respectively. The C=O stretching vibration peak at 1665 cm^−1^ and the peaks at 2933 cm^−1^ and 2785 cm^−1^ corresponded to the stretching vibration of the methylene groups [27]. The absorption peak at about 1416 cm^−1^ resulted from the stretching vibration of C-N in the PAAm hydrogel, while the absorption bands at 1190 cm^−1^ and 1123 cm^−1^ corresponded to the bending vibrations of C-H. Compared with the pure PAAm hydrogel, the chromogenic indicator after BCG adsorption had similar infrared spectra, in which the enhancement of the 1321 cm^−1^ absorption peak was due to the C-O of the phenol in BCG. Nevertheless, after the adsorption of BCG, the C=O stretch peak observed at 1665 cm^−1^ of the PAAm hydrogel shifted slightly to 1660 cm^−1^, and it might be caused by the hydrogen bond formation between BCG and the PAAm hydrogel [28]. The FTIR spectra results indicated that BCG was adsorbed on the PAAm hydrogel through physical interaction, neither changing the chemical structure of the PAAm hydrogel nor forming chemical interactions between them.

#### 3.2.2. Thermal Stability

**Figure 3:**
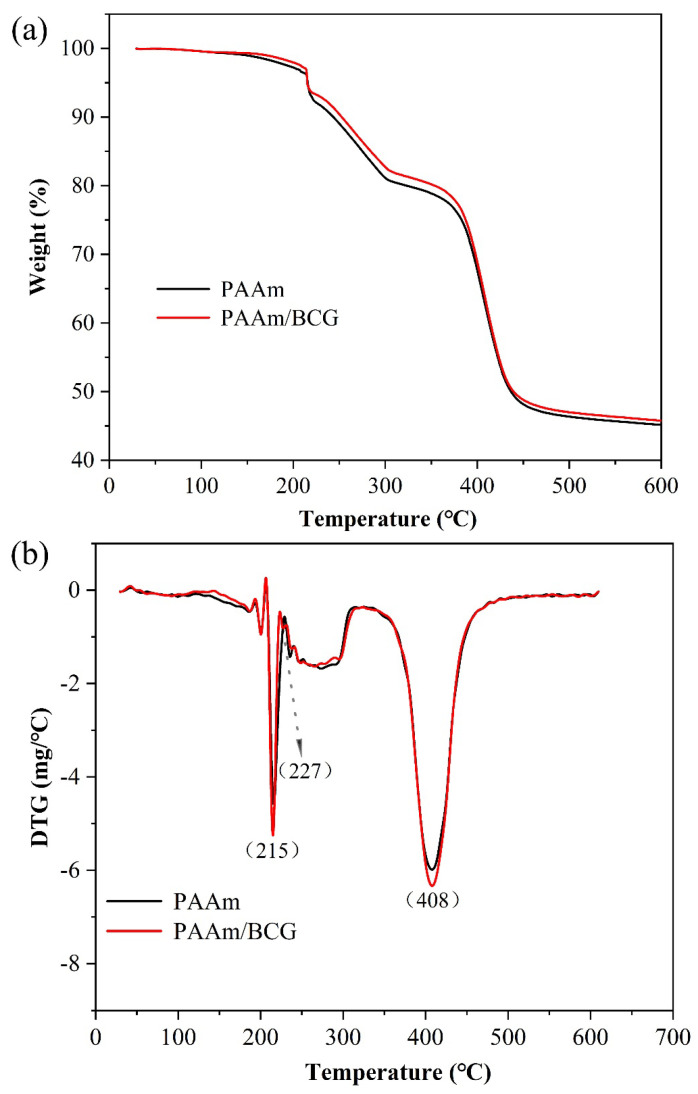
(**a**) TGA and (**b**) DTG thermograms of the PAAm hydrogel and PAAm/BCG indicator.

The TGA thermogram showed the weight loss rate (%), and the derivative thermogravimetric (DTG) thermogram (a derived form of TGA) showed the maximum decomposition temperature (T_max_) in each thermal decomposition [24]. Both the pure and the BCG-adsorbed PAAm hydrogel showed three steps of thermal decomposition (Figure 3). Firstly, the weight loss was observed at around 100 ℃ with a rate of about 2%, which was due to the volatilization of small-molecule solvents, water that had not been removed from the hydrogel system, and water adsorbed by the polymer to the outside [29]. The second and most significant weight loss occurred around 210–310 °C due to cleavage of the PAAm hydrogel matrix. In the third stage, owing to substituent (-NH_2_) decomposition, the weight of the sample was reduced by about 34% at 350–500 °C, and the decomposition rate was rapid [30]. Finally, the residual char content at 600 °C was 45.8% for both samples. In addition, at around 227 °C, BCG gradually fell off from the indicator and volatilized. After that, some T_max_ values were shifted to a higher temperature, which was due to the fact that BCG was a heat-resistant hydrophobic compound that could improve the thermal stability of the PAAm hydrogel. In addition, the weights of the PAAm hydrogel sample and the PAAm/BCG indicator sample decreased by 0.0177% and 0.0074%, respectively, from 124 °C to 126 °C, indicating that the amount of residual acrylamide monomer in the samples was very low. The residual acrylamide monomer content in PAAm/BCG was less than that in PAAm, which might be due to the polymerization reaction between the double bond of the acrylamide molecule and BCG.

#### 3.2.3. MC, WS, and WA Analysis

The MC, WS, and WA of the indicator are presented in Table 1. The MC of pure PAAm hydrogel was 50.26%. The appropriate MC indicated that the indicator had a certain moisture resistance, meanwhile, it was conducive to the color reaction of the indicator. Moreover, the addition of BCG decreased the MC of the indicator slightly. This was due to BCG being a polyphenolic compound, and the hydrophobic group of pigment decreased the MC of the indicator structure. On the other hand, the bubble enlarged the size of the holes inside the hydrogel and could be attributed to the higher MC, which facilitated the sensing indicator to be more sensitive to volatile compounds [31]. It was worth noting that the 3D network structure of the PAAm hydrogel could effectively inhibit the transformation of fish from bound water to free water in the myofibrillar protein network, which contributed to maintaining the freshness of fish [32].

The WS represented the physical barrier and water resistance properties of the indication indicator inside the package [33]. The WS of the neat PAAm hydrogel was only 14.26%, which showed that the PAAm hydrogel sample had good water holding capacity and swell ability. This might be attributed to the relatively large crosslinking density of the polymer matrix. In addition, the significant decrease in WS in the PAAm hydrogel/BCG tag (*p* < 0.05) was also due to the hydrophobicity of BCG [34].

The WA reflected the ability of the indicator to absorb water during storage and distribution of the packaged product, and had a significant effect on the efficiency of the color response. That is, the higher the WA value, the faster the pigment was released [35]. The WA of the PAAm hydrogel was 132.38%, and adding BCG to the PAAm hydrogel dropped the WA to 103.02%, owing to the hydrophobic properties of BCG. Another reason might be due to the reduction of crosslinking density, reducing the porosity and water retention capacity of the indicator [36].

#### 3.2.4. Release of BCG

**Figure 4:**
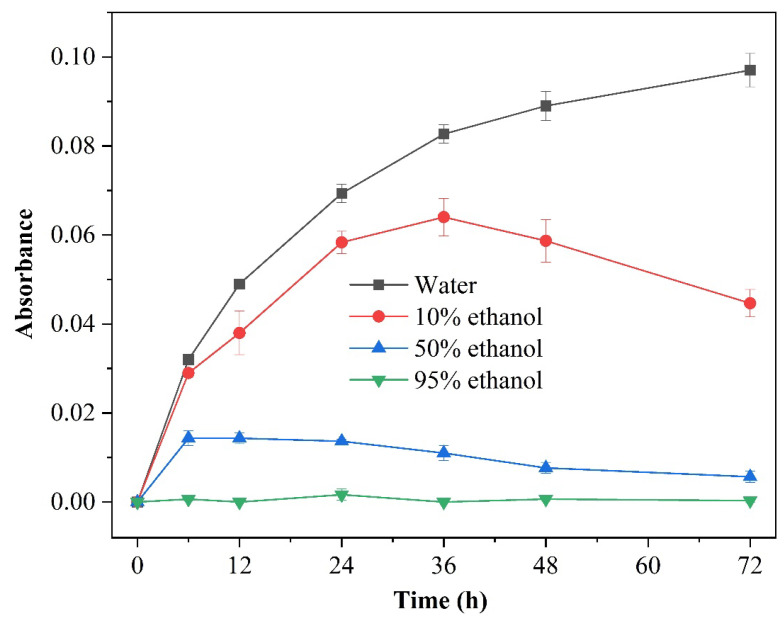
The release profile of BCG from the PAAm/BCG indicator in various food stimulants.

In general, the release rate of reactive pigments depends on a variety of factors, including the solubility of the internal pigment, the WA of the polymer, and the rate of diffusion of the pigment through the hydrogel matrix [37], As shown in Figure 4, the release of BCG was highly dependent on the type of food simulant solutions used. Among them, the largest amount in pure water, followed by 10%, 50%, and 95% alcohol solutions. The absorbance in the food simulant solution with moderate alcohol content (10% and 50%) showed a downward trend, which was due to the dissolution of the PAAm hydrogel making the simulant solution tend to be acidic, and the maximum absorbance value shifted from 615 nm to 445 nm, as confirmed by the UV-vis spectra analysis results. Furthermore, the absorbance of the 95% alcohol food simulant was essentially unchanged, possibly due to the increase in crosslink density when the hydrogel matrix was exposed to high alcohol concentrations. Therefore, although BCG was an alcohol-soluble pigment with fast release in high-concentration alcohol solutions, it made up for the deficiencies in the application of indicators in fish products, owing to the properties of the hydrogel matrix.

### 3.3. Color Response Efficiency

**Figure 5:**
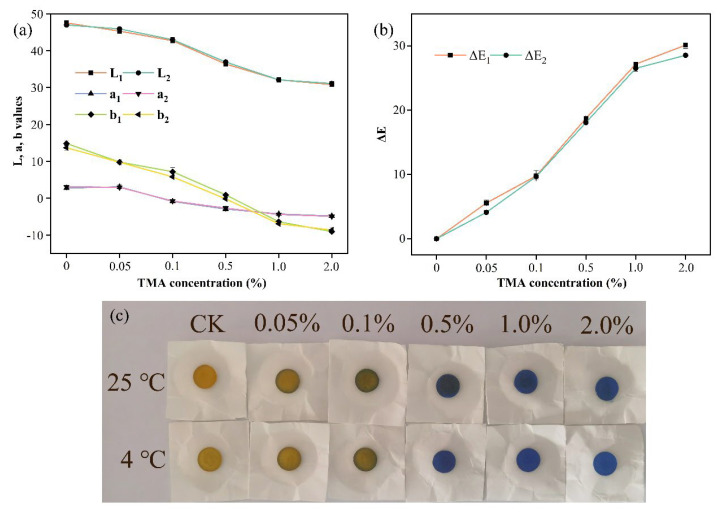
(**a**) L, a, and b, (**b**) Δ*E* values, and (**c**) color responsive styles in different concentrations of TMA solutions at 25 °C and 4 °C.

As shown in Table 2, the PAAm/BCG indicator revealed a significant color change at pH 3.0–8.0. Specifically, the indicator acted as a visual sensing means of yellow, light green, and dark green in turn at pH 3.0–5.0. With the pH raised to 6.0, the indicator exhibited an aquamarine color, and it showed a blue color when exposed to pH 7.0–8.0. This was similar to the result of Figure 5b, indicating that the PAAm hydrogel matrix had little effect on the color response of BCG. In addition, the color change of the PAAm/BCG indicator was also observed in the tristimulus color values. The L-value (lightness) was the highest at pH 3.0 and lowest at pH 8.0, showing that the indicator was the brightest and darkest in acidic and basic environments, respectively. The a-value (greenness/redness) decreased to pH 5.0, then increased slightly as the pH increased to 8.0, signifying that the indicator turned greenness overall with increasing pH values. Similarly, the b-value (blueness/yellowness) decreased obviously as the pH increased to 8.0, demonstrating a high intensity of blueness of the indicator in alkaline conditions. The total color difference (Δ*E*) increased with pH and was generally inversely proportional to a- and b- values. Markedly higher Δ*E* values of the indicator at pH 3.0–5.0 and then Δ*E* stabilized at pH 6.0–8.0 meant that the indicator showed clear color changes when encountering basic compounds.

In practical applications for intelligent packaging, the response of the indicator to the color changed after exposure to steam must be unique and rapid. Here, ammonia, dimethylamine (DMA), TMA and other mixtures of mono- and polyamines could be produced by the metabolic breakdown of amino acids during fish products spoilage [38]. The color response of the indicator to different concentrations of TMA solutions was determined to validate the sensitivity to pH changes (Figure 5). The concentration of TMA gas in the airtight container was proportional to the concentration of TMA solution [39], inversely proportional to the tristimulus color values of the indicator, and positive to the Δ*E* value related. The large change in a-value indicated that the indicator had a significant visual effect when encountering the fish spoilage gas, and the degree of spoilage could be judged according to the depth of the color. In addition, the tristimulus color values and visual effects of indicators at room temperature (25 °C) and refrigerated (4 °C) were not significantly different.

### 3.4. Packaging Test

#### 3.4.1. Monitoring Freshness of Japanese Sea Bass (*Lateolabrax japonicus*)

During the storage, processing, and transportation of fish, free amino acids and trimethylamine-N-oxide underwent conversion into biogenic amines; the adenine nucleotides gradually degraded to form inosine and xanthine, and sugars and lactate salts were metabolized to generate various other organic acids [40,41]. Moreover, the specific decomposition led to the production of alkaline nitrogenous substances, such as ammonia and amines. These substances were volatile, and their content increased with the degradation of amino acids, particularly methionine and tyrosine. Hence, microbial spoilage and biochemical reactions propelled the accumulation of volatile amines and then contributed to the increased pH of the packaging headspace gradually [42]. Noteworthy, the alternation in the color of the indicator could display the onset of the decay process visually. However, various indicators of fish meat spoilage were delayed during the refrigeration period, especially the metabolism of most spoilage bacteria [43]. Therefore, refrigeration is currently a significant means of preserving raw fish slices [44]. The packaging test of this article was also carried out under refrigerated temperature.

**Figure 6:**
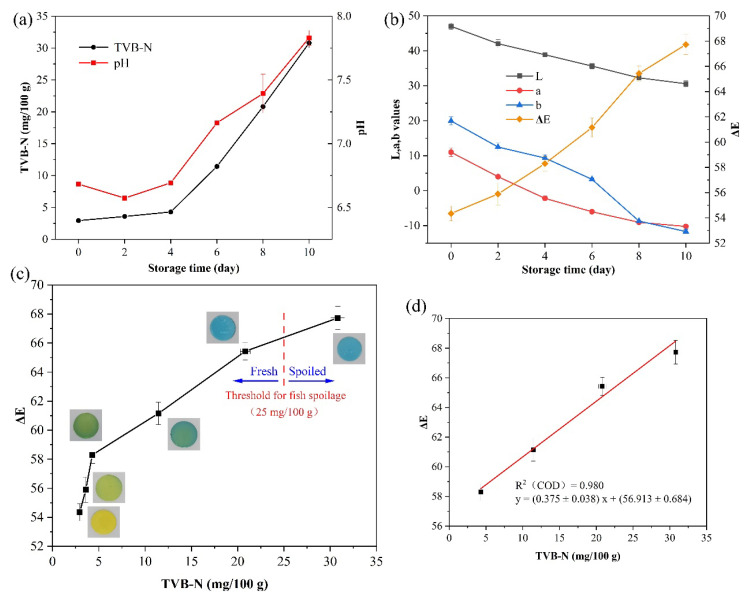
Changes in (**a**) the TVB-N levels and pH, (**b**) L, a, b, and Δ*E* values during storage of sea bass at 4 °C. (**c**) Δ*E* values of the indicator in response to different amounts of TVB-N. (**d**) Linear fitting of Δ*E* values to TVB-N.

The pH values and TVB-N levels for sea bass samples stored at 4 °C for 10 days are provided in Figure 6, as well as the visual color, tristimulus color, and Δ*E* values changed by the indicator. Among them, Figure 6a shows that the initial pH value of sea bass was 6.68 ± 0.02 on day 0, which indicated the fresh state of the fish samples. On day 2, the pH value of the fish simple dropped to 6.57 ± 0.01 and then gradually increased over the next few days. The decrease in pH was caused by the breakdown of muscle glycogen in the process of fish to produce lactic acid and other acids, after which the increase in pH was due to the progression of alkalization resulting from the release of essential products of protein breakdown throughout the postmortem change. These results were consistent with the previous studies [45].

The TVB-N level was widely regarded as the key index for reflecting fish spoilage. A value of 25 mg/100 g was proposed as the highest acceptable level [46]. The initial TVB-N level of the fish sample was 2.94 ± 0.2 mg/100 g, indicating that the sample was fresh. Subsequently, TVB-N increased slowly before day 4 and increased rapidly after day 4. It was attributed to microbial activity and protein degradation by autolyzing enzymes, resulting in amino acid deamination and volatile base accumulation [47]. On day 8, the TVB-N level of sea bass samples reached 20.81 ± 0.54 mg/100 g, which was close to the spoilage threshold. In addition, the TVB-N level reached 30.80 ± 0.75 mg/l00 g on day 10, which was higher than 25 mg/100 g, indicating that the sea bass at this time had been spoiled and inedible.

As shown in Figure 6c, due to gradually increased volatile amines in the headspace, the color of the visual indicator also changed from yellow to green to blue. Therefore, the tristimulus color values also showed corresponding trends (Figure 6b). The L-value was the highest on day 0 and lowest on day 10, and showed a downtrend, indicating that the color brightness of the indicator would gradually become darker as the fish spoiled. Similarly, the a-value and the b-value also showed a downtrend and the variation range of the b-value was more than the a-value, showing that the indicator tended to turn green and blue, respectively. The Δ*E* values of the indicator increased during storage at 4 °C, and the uptrend was more pronounced after day 6.

The results of our study confirmed a valid correspondence among pH increment, TVB-N generation, and visual color of the PAAm/BCG indicator. Therefore, the change in color of the indicator depended on the production of TVB-N and was more pronounced with the increase in TVB-N levels. Figure 6c plotted the Δ*E* value against TVB-N concentration in the headspace of fish packaging, and showed the visual specific color change of the indicator and the threshold for fish spoilage during storage.

#### 3.4.2. Feasibility Analysis of Models and Data

The correlation between the Δ*E* value of the indicator and the TVB-N of fish during storage at 4 °C was evaluated by the R parameter, implying the strength of the correlation. Additionally, linear polynomial models and R^2^ (the precision of the model) were confirmed after day 4 (Figure 6d). The correlation between the Δ*E* of the indicator and the TVB-N of sea bass over the range of 4.29 ± 0.13 to 30.80 ± 0.75 mg/100 g (R^2^ = 0.98, y = (0.375 ± 0.038) x + 56.913 ± 0.684) was high and positive. In addition, the values of root mean square error (RMSE), sum of squares error (SSE), and Akaike Information Criterion (AIC) are 0.51, 1.03, and 4.57, respectively. It indicated that the fitting effect of the model is good. However, the slightly high AIC value is also a result of the insufficient sample size.

### 3.5. The Advantages and Disadvantages of the PAAm Hydrogel/BCG Indicator

The biggest advantage of the PAAm hydrogel/BCG indicator was a relatively simple and economical preparation process. The indicator was prepared using the sol-gel method. The weak acidity of the acrylamide monomer aqueous solution made the indicator appear yellow after preparation and molding. Therefore, it was not necessary to consider the color status of the indicator before use, eliminating the cumbersome process of adjusting the pH of the pre-liquid, and it was more advantageous than some experiments that require acid toning [48,49]. Furthermore, due to the proper ratio of monomer content to water, the use of a crosslinking agent was avoided. The sol could be turned into a gel by adding a thermal initiator and heating, forming process only 25 min, which was more time-saving than other kings of literature [50,51,52]. However, the PAAm hydrogel/BCG indicator required experiments related to recycling to achieve economic sustainability. Moreover, the protection measures of the indicator in practical applications should be further considered.

## 4. Conclusions

To sum up, a novel freshness indicator was prepared by adsorbing BCG to the PAAm hydrogel and exhibited noticeable color changes relating to fish freshness during storage. The indicator could be quickly prepared by injection molding, which was suitable for mass production. Benefiting from the PAAm hydrogel matrix (MC = 50.26%, WS = 14.26% and WA = 132.38%), the indicator (MC = 43.45%, WS = 9.60% and WA = 103.02%) had good moisture resistance, which contributed to avoiding moisture, affecting the measurement accuracy of the indicator. Both FTIR analysis and TGA indicated that the PAAm hydrogel had excellent compatibility with BCG. The indicator showed that the color changed from yellow to blue at pH 3.0–8.0 clearly, and the hydrogel matrix had little effect on the color response efficiency of BCG. It was worth emphasizing that Δ*E* had a highly linear relationship with TVB-N (R^2^ = 0.98, y = (0.375 ± 0.038) x + 56.913 ± 0.684), and the fitting effect of this model was good (RMSE = 0.51, SSE = 1.03, and AIC = 4.57). However, there were still some shortcomings in this article, such as the lack of evaluation of the biodegradation ability of PAAm hydrogel and the inconspicuous color change of the indicator when approaching the spoilage threshold. Overall, based on the pH-sensitive visual indication system, different color changes were presented, which were easier for the human eye to distinguish and facilitated the monitoring of fish freshness. Compared to traditional freshness detection techniques, this method was more acceptable, convenient, and cost-effective. Therefore, the PAAm hydrogel/BCG indicator as a rapid and simple intelligent approach indicates its feasibility as a real-time monitoring of freshness for potential prospects.

## Figures and Tables

**Table 1:** Moisture content, water solubility and water absorption of PAAm hydrogel samples and PAAm hydrogel/BCG samples.

Samples	MC (%)	WS (%)	WA (%)
PAAm hydrogel	50.26 ± 0.44 ^a^	14.26 ± 0.43 ^a^	132.38 ± 9.22 ^a^
PAAm hydrogel/BCG	43.45 ± 1.08 ^b^	9.6 ± 2.8 ^b^	103.02 ± 13.21 ^b^

The values are presented as a mean ± standard deviation. The values of the same column, followed by different letters, are significantly different (*p* < 0.05).

**Table 2:** The color change of the PAAm/BCG indicator in response to different pH buffer solutions and variations in color parameters.

pH							
	Control	3	4	5	6	7	8
*L*	96.52	36.32 ± 1.46 ^c^	35.69 ± 0.82 ^bc^	33.37 ± 0.24 ^ab^	31.57 ± 2.47 ^a^	31.48 ± 0.47 ^a^	31.23 ± 0.28 ^a^
*a*	−0.24	1.42 ± 0.10 ^e^	−0.48 ± 0.19 ^d^	−3.46 ± 0.31 ^a^	−2.38 ± 0.17 ^b^	−2.01 ± 0.29 ^bc^	−1.73 ± 0.11 ^c^
*b*	0.46	7.27 ± 1.07 ^c^	6.75 ± 0.39 ^c^	−0.61 ± 0.38 ^b^	−1.06 ± 0.64 ^b^	−4.81 ± 0.69 ^a^	−5.59 ± 0.42 ^a^
Δ*E*	0	60.59 ± 1.56 ^a^	61.13 ± 0.85 ^a^	63.21 ± 0.24 ^ab^	64.98 ± 2.4 ^b^	65.25 ± 0.44 ^b^	65.56 ± 0.2 ^b^
Color changes		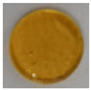	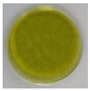	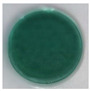	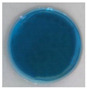	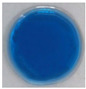	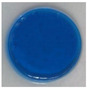

The values are presented as a mean ± standard deviation. The values of the same column, followed by different letters, are significantly different (*p* < 0.05).

## Data Availability

The datasets generated during and/or analyzed during the current study are available from the corresponding author on reasonable request.
